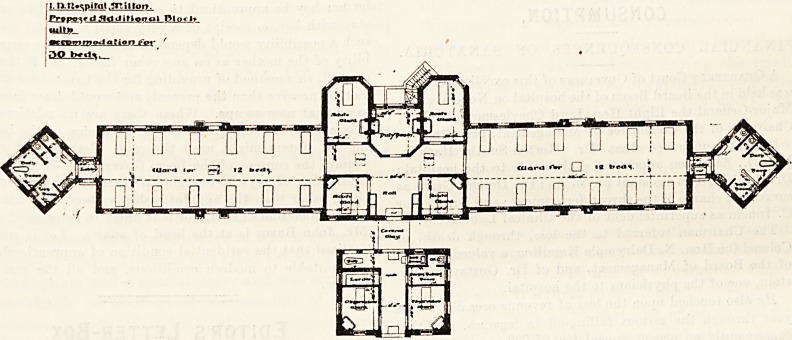# New Block at the Milton Hospital for Infectious Diseases, Portsmouth

**Published:** 1906-12-01

**Authors:** 


### NEW BLOCK AT THE MILTON HOSPITAL FOR INFECTIOUS DISEASES, PORTSMOUTH.

A plot of land three acres in extent was recently added to the old site, and on this plot has been erected a well-arranged block for infectious diseases, and also a small block for cases when under special observation. Excepting the heating chamber, placed in the basement, the buildings have ground floors only.

The main block is 255 feet long, and is composed of a centre and two wings. The centre is approached from a covered-way which serves the double purpose of a porch and a means of communication between the main block and the subsidiary one. Next this porch is the hall, having a single-bedded ward for acute cases on each hand, and beyond these is the wide corridor of communication between the large wards. Beyond this corridor is the nurses' duty-room and two single-bedded wards for acute cases. These rooms have bay windows, and, as the beds are placed in the bays, there will be a fair amount of cross-ventilation; but an extra window on each outer wall would have been an improvement. The other single-bedded wards have each two windows on the side facing the observation block, but they are without cross-ventilation as far as windows are concerned; and this fault might have been to some extent obviated had this part of the centre been thrown outwards for five feet, when a window could have been placed in the side and a current of air obtained across the angle.

The main wards are 72 feet long, 26 feet wide, and 14 feet high. Each bed has therefor 12 feet of wall-space, 156 square feet of floor-space, and nearly 2,200 cubic feet of air-space; and these dimensions are quite sufficient when, as in this case, the cross-ventilation has been secured. Every bed has a window on both sides, and the windows have fanlights which are hinged so as to fall inwards on glass hoppers. At the head of each bed is also a fresh-air inlet. The sanitary annexes are at the extreme ends of the wards. They are arranged in a compact manner and are properly cut off from the block.

The observation block, as already said, is connected with the main block by the porch. It is divided into equal parts by a wide passage, open at both ends. It contains two single-bedded observation wards, a nurses' robing room, a bathroom, larder, etc.

The elevations of the new blocks are built of local bricks, with Portland stone dressings, and the roofs are slated. The ward floors are of pitch pine; but those of the sanitary annexe are of asphalt, as also is the wide passage in the observation block. The walls of the sanitary annexes are lined with glazed bricks, while the ward walls and ceilings are finished in granitic plaster. In all cases angles have been rounded off.

The heating is by open stoves assisted by steam radiators. The lighting is electric, and each bed is supplied with its own bracket-lamp for use when necessary. Gas is also used as an auxiliary and for purposes of cooking. The sanitary fittings seem extremely good and are of quite modern construction.

Exclusive of electric light fittings the cost was almost £6,000, and, as the total accommodation is for thirty beds, the cost per bed exceeded £200. The architect was Mr. Philip Murch, the borough engineer; the contractor for the building was Mr. Colterup, of Portsmouth; and the electric engineers were Messrs. Southey, of Southsea.

## Figures and Tables

**Figure f1:**